# Exploring the evolution of engagement between academic public health researchers and decision-makers: from initiation to dissolution

**DOI:** 10.1186/s12961-019-0516-0

**Published:** 2020-02-10

**Authors:** Nasreen S. Jessani, Akshara Valmeekanathan, Carly Babcock, Brenton Ling, Melissa A. Davey-Rothwell, David R. Holtgrave

**Affiliations:** 1 Bloomberg School of Public Health, Johns Hopkins University, Baltimore, United States of America; 2 Center for Evidence Based Health Care, Department of Global Health, Stellenbosch University, Cape Town, South Africa; 3 Africa Centre for Evidence, Faculty of Humanities, University of Johannesburg, Johannesburg, South Africa; 4 School of Public Health, University at Albany, State University of New York, Rensselaer, NY USA

**Keywords:** Government, academia, relationship, evidence-informed decision-making, evolution, resilience, network

## Abstract

**Context:**

Relationships between researchers and decision-makers have demonstrated positive potential to influence research, policy and practice. Over time, interest in better understanding the relationships between the two parties has grown as demonstrated by a plethora of studies globally. However, what remains elusive is the evolution of these vital relationships and what can be learned from them with respect to advancing evidence-informed decision-making. We therefore explored the nuances around the initiation, maintenance and dissolution of academic–government relationships.

**Methods:**

We conducted in-depth interviews with 52 faculty at one school of public health and 24 government decision-makers at city, state, federal and global levels. Interviews were transcribed and coded deductively and inductively using Atlas.Ti. Responses across codes and respondents were extracted into an Excel matrix and compared in order to identify key themes.

**Findings:**

Eight key drivers to engagement were identified, namely (1) decision-maker research needs, (2) learning, (3) access to resources, (4) student opportunities, (5) capacity strengthening, (6) strategic positioning, (7) institutional conditionalities, and (8) funder conditionalities. There were several elements that enabled initiation of relationships, including the role of faculty members in the decision-making process, individual attributes and reputation, institutional reputation, social capital, and the role of funders. Maintenance of partnerships was dependent on factors such as synergistic collaboration (i.e. both benefit), mutual trust, contractual issues and funding. Dissolution of relationships resulted from champions changing/leaving positions, engagement in transactional relationships, or limited mutual trust and respect.

**Conclusions:**

As universities and government agencies establish relationships and utilise opportunities to share ideas, envision change together, and leverage their collaborations to use evidence to inform decision-making, a new modus operandi becomes possible. Embracing the individual, institutional, networked and systems dynamics of relationships can lead to new practices, alternate approaches and transformative change. Government agencies, schools of public health and higher education institutions more broadly, should pay deliberate attention to identifying and managing the various drivers, enablers and disablers for relationship initiation and resilience in order to promote more evidence-informed decision-making.

## Introduction

Historically, universities have offered faculty the opportunity to pursue a career of exploratory thought and discovery. However, some see this privilege as disconnected from the realities of the surrounding environment and have attached a reputation of universities as ‘ivory towers.’ Recognition that universities are inextricably embedded within a locally and globally networked environment [1, 2] has brought with it an expectation by society and a desire by academia to engage with these networks. Furthermore, universities are increasingly being seen as agents of social, economic and technological change in today’s knowledge economy [3–5]. This is reflected in research funding agencies (including governments) requiring evidence of social returns on their investments in the forms of ‘impacts’ or ‘benefits’ to society [6] . The Research Excellence Framework in the United Kingdom, for instance, launched in 2014 by four United Kingdom higher education funding bodies, defined impact as “*an effect on, change or benefit to the economy, society, culture, public policy or services, health, the environment or quality of life, beyond academia*” [7].

In parallel, government departments and agencies are expected to make decisions that impact the same complex environments that universities seek to contribute research and knowledge to. The impetus for such agencies to utilise evidence to inform their policies is apparent globally, ranging from high-income countries like the United Kingdom [7], Australia [8] and Canada [9] to low- and middle-income countries such as Kenya [10] and South Africa [11], amongst others. This increasing importance in the role that evidence plays in decision-making presents an opportunity for researchers and academics to work with decision-makers in bringing this evidence to bear on the decision-making process. These engagement opportunities in turn underscore the importance of relationships in the decision-making process.

Similar to other scholars [12], we use ‘engagement’ here as a broad, all-encompassing term that captures the various ways in which researchers and decision-makers interact. This could include collaborative research, research–practice partnerships, science advice, research priority-setting, policy priority-setting, research dissemination and use, technical assistance, policy advice, subject matter expertise, training, etc. These numerous ways of interacting include common elements along with those that are more context specific, therefore creating points of influence when it comes to setting up successful engagement opportunities for public health faculty and decision-makers. Identifying those elements that are pervasive and perhaps more stable in nature may provide ways to potentially mitigate risk of relationship dissolution as well as support initiation and maintenance. A focus on only one type of engagement would not only narrow our understanding of the nuances of each type of engagement but would also negate the recognition of the variety of ways in which colleagues engage with each other.

### Researcher–government engagement

Scholars have noted that frequent interactions between researchers and decision-makers is likely to yield research that is relevant for informing policy and practice priorities [13–19] and therefore more likely to be considered during decision-making [20]. Furthermore, such engagement fosters mutual understanding of the environmental realities that researchers as well as decision-makers have to contend with [15], hence facilitating the positioning of research findings accordingly [13].

However, engagement does not come without its challenges; systematic reviews on facilitators and barriers to engagement between researchers and decision-makers document these challenges extensively [20–23]. Interest in better understanding the relationships between academic researchers and their stakeholders has grown with literature demonstrating a plethora of such studies globally. We found papers examining relationships between academia and industry [21–27], health services [28–33], non-profit organisations [34], boundary organisations [35], and patients, communities and the public [33, 36], amongst others. Similarly, studies focusing on government and their stakeholders such as think tanks [37] and advocacy organisations [38, 39] also contribute to this dynamic and evolving stream of inquiry.

However, studies focused on examining direct relationships between academia and government, while existent [16, 21, 37, 39–41], are varied in their focus. While indicators for assessing collaborations have been suggested [42], we assert that the focus is narrow. Assessment of how engagement, in a broader sense, begins and evolves and what practices could be put in place to support them as well as mitigate anticipated challenges could be strengthened. Reed et al. [43] call for more research on “*assessing pathways to policy impacts that can provide feedback to researchers and policy-makers to enhance impact, whilst also providing reliable evidence when far-reaching and significant impacts occur*”.

In our quest to further understand the complexities of these pathways and the relationships between academic researchers and government decision-makers, we were particularly interested in links between schools of public health (SPHs) and government. Our exploration yielded some studies that have endeavoured to map the relationships and networks between SPHs and government agencies, for instance in Kenya [44], Australia [45] and the United States of America [46, 47]. However, what remains elusive is the evolution of these vital networks of epistemic communities, the nature of the relationships with government, and what can be learned from them with respect to advancing evidence-informed decision-making (EIDM) [22, 48].

As other scholars note, the relationships between researchers and decision-makers are complex, dynamic and constantly evolving as a result of changing circumstances; they can thus be conceptualised as “*social learning processes*” [43, 49]. The evolution of relationships and engagement opportunities between academia and government in terms of how they are initiated (by whom and for what reasons), how they are maintained (with what resources and supports), and why they dissolve or terminate are therefore important to understand so as to be strategic about such engagements, allocate relevant resources towards them, and finally to anticipate and mitigate potential problems.

### Schools of public health and the United States context

At the time of this paper, there were 66 accredited SPHs in the United States [50]. The importance of strengthening relationships between SPHs and government public health agencies in the United States was emphasised over 30 years ago as documented in the Institute of Medicine *Future of Public Health Report* [51], *The Public Health Faculty/ Agency Forum (PHFAF)* report [52], and the Pew Commission report [53]. Few studies have sought to review the impact of the three reports mentioned above, on the linkages between SPHs and USA health agencies [54, 55].

The Council on Education in Public Health (CEPH) is the national accreditation body within the United States that oversees SPHs as well as public health programmes outside schools of public health [50, 56]. Additionally, CEPH has the capacity to accredit international schools of public health. CEPH has a parallel agency in the European context called Agency for Public Health Accreditation [57]. Both agencies have professional associations that represent accredited schools and programmes of public health and services to train and build capacity of public health professionals [58, 59].

### Context of Johns Hopkins Bloomberg School of Public Health

Established in 1916, the Johns Hopkins Bloomberg School of Public Health (JHSPH) is the largest school of public health in the world, comprising 10 departments, over 70 centres and institutes, approximately 700 full-time faculty, and 2650 students [60]. The teaching and research across departments encompass five core areas of public health previously articulated by the Association of Schools and Programs in Public Health [58]: Health Policy and Management, Health Behavior and Society, Biostatistics, Epidemiology, and Environmental health. The additional five departments include International Health, Microbiology and Immunology, Mental Health, and Population, Family and Reproductive Health. Located in Baltimore, JHSPH enjoys close proximity to various Baltimore City, Maryland State, and United States Federal government agencies. Furthermore, international faculty, students and research projects provide opportunity to engage with governments globally.

### Paper aims

While understanding that ‘what works’, ‘why’ and ‘how’ is influenced by context – the individuals interacting, the culture of the organisations in collaboration, external environmental factors [15, 40, 41, 61–66] ideas, networks and events anticipated and unexpected [67] – we explored the evolution of relationships between academic faculty at one SPH in the United States – JHSPH – and government decision-makers. By focusing on initiation, maintenance and dissolution of these relationships, instead of treating engagement as a stable concept, this study fills a gap in existing engagement literature.

Within this exploration we had two main aims:

1. Understand drivers as well as enablers for initiation, maintenance and dissolution of academic–government engagements, and within these seek to uncover key characteristics of these relationships that are influential and stable over time. The explorations require consideration of both – the academic as well as the government – perspectives and reflections.
2. Utilise the findings from this study to contribute to deliberations within SPHs, and even universities as a whole, about how to be more strategic about academic–decision-maker engagement, allocate relevant resources towards them, and to anticipate and mitigate potential problems.

## Methods

The data for this paper emerged from Phase II of a larger study focused on faculty JHSPH and government decision-makers at city, state, federal and global levels. Phase I (Jun–Dec 2016) consisted of network mapping and analysis of academic faculty relationships with government decision-makers [47] and therefore served as the platform for in-depth interrogation of the network’s development with a subsample of the respondents in Phase II.

### Respondent Selection

Selection criteria of full-time faculty for Phase I of the study have been described extensively elsewhere [47]. For Phase II, a subsample of the 211 respondents from Phase I were chosen for semi-structured interviews based on whether they were highly engaged, namely Faculty who had five or more contacts with decision-makers at any one government level and/or in the top 10 percentile of those with the most connections across all four government levels (*n* = 49), or non-engaged, namely Faculty with zero or one contacts with decision-makers (*n* = 57).

In addition, decision-makers who were mentioned by two or more faculty (*n* = 92) during the network mapping in Phase I were shortlisted. This was due to an underlying assumption that decision-makers identified multiple times in Phase I interviews would likely have more to share on their various interactions and relationships with faculty. A decision-maker, for this study, was defined as someone who plays a key role in the administration (and leadership) of an organisation, in a position to make decisions or exert influence in a decision-making situation.

### Instrument design

Two separate semi-structured interview guides were created – one for faculty members and another for decision-makers. The instruments were adapted from a previous study [68, 69] and revised in light of results from Phase I [70]. Colleagues from the Schools of Medicine and Nursing who were not eligible for the study also reviewed the instrument in order to ensure applicability.

Faculty questions explored reasons for engagement or non-engagement (drivers), individual and institutional factors that affect engagement (enablers), the role of researchers in bringing evidence to bear on decision-making, experiences engaging with decision-makers, circumstances that lead to initiation, maintenance and dissolution of relationships, reflections on SPH initiatives in addressing ‘practice’ relevant opportunities, and advice for peers and SPH leadership. We also collected socio-demographic information, including age, sex and academic qualifications along with some organisational information (departmental affiliation, academic position, leadership position) in order to contextualise any variation in responses. For the decision-makers, questions revolved around their reasons for engaging with academics, what affects their choice of institution and/or academic faculty, their engagement experiences, the nature of their relationships with faculty, including information on their initiation, maintenance and dissolution, and any advice they had for faculty as well as SPHs more generally.

### Data collection and analysis

Between November 1, 2017, and February 5, 2018, eligible faculty and decision-makers were contacted a maximum of two times to respond to the initial invitation. All willing respondents were accommodated for a semi-structured interview within this time frame. Interviews were conducted either in person, via Skype or by phone and lasted between 30 and 75 min. The flexibility in mode was meant to accommodate geographic spread of respondent locations as well as time differences. All interviews were audio-recorded with verbal participant consent, and transcribed verbatim. In order to enhance credibility of the data, the study team reviewed the transcripts, which were examined collectively to discuss inconsistencies and potential variations in interpretations. Each transcript was also supplemented with notes made during as well as immediately after the interview, capturing respondent attitudes and emotions about the topic. Transcripts were read and re-read to increase familiarity with the vast dataset. Emerging findings were shared and discussed regularly by the study team in an attempt to discern any personal biases and determine data saturation.

Transcribed interviews were imported into ATLAS.ti 8 [71] for analysis and codebook development. Themes central to interview questions served as the initial guide for codebook development (deductive coding). The codebook was refined with emerging themes as a result of inductive analysis of the data [72]. A sample of transcripts were co-coded by three members of the study team to establish inter-coder reliability. Nuanced interpretations were discussed and documented, especially when different interpretations of the same quote arose. Common codes, which occasionally were given different names, were collapsed into one. Differences in coding were discussed, and codes further clarified to minimise misinterpretations or errors in coding. This resulted in the first draft of the codebook, which was revised iteratively as more transcripts were reviewed and until no new codes were generated. The final codebook comprised of a full description of each code, detailed notes on when to use and when not to use each code, and an example transcript text for code utilisation. Eighteen primary codes were finalised for decision-maker interviews and 22 for faculty interviews, each with several sub-codes. The final codebook was imported to ATLAS.ti and applied to all transcripts.

The code extraction template in Excel comprised of one row per respondent and one column per code and transcript data was inserted verbatim into the corresponding cell in the matrix. Responses across codes and respondents were compared across the matrix and connections were mapped. Additional themes and sub-themes, where relevant, were then generated from the dataset. Both the original research objectives as well as new ideas generated inductively from the dataset guided this process. Patterns observed within themes, across themes as well as respondents linked to different sets of themes were further examined through structured memos. These memos also included illustrative respondent quotes, which later contributed towards the writing of this paper.

## Results

### Participant overview

The overall faculty response rate was 70% (74/106), with a 73% (36/49) response rate amongst highly engaged faculty and 67% (38/57) response rate amongst those categorised as non-engaged. We interviewed 52/70 (75%) faculty who indicated willingness to participate (36 in person, 12 over video Skype and 4 by telephone) as shown in Table 1. Given the small number of Professors Emeritus as well as Professors of Practice, we have combined them and contracted their titles to ‘Professor’ in order to minimise identification. In order to further enhance anonymity without diluting respondent diversity or content of the quotes used, we have replaced SPH department names with randomly assigned numbers.

**Table 1:** Overview of academic faculty respondents

	Number of eligible Faculty	Respondents (% of total)	Sex		Track			Seniority	
			M	F	Professor	Scientist	Other	Associate and above	Assistant and below
Total (N)	106	52 (49)	20	32	27	17	8	29	23

We had respondents from all departments with fairly equal distribution across seniority as well as academic tracks. Fifteen respondents held leadership or administrative management positions, with some holding both.

Of the 92 eligible decision-makers, 23 (25%) had either relocated to another part of the government system, left their respective agencies or had no publicly accessible contact information. Of those we were able to contact, we had a positive response rate of 45% (31/69). We interviewed 24/31 (77%) decision-makers (15 over the phone and 9 via Skype). Decision-makers from all four government levels were represented with the majority coming from the State level (Table 2). The relative seniority of these positions varied across agencies. Experiences reflecting the multi-sectoral engagements between faculty and decision-makers, spanning a range of agencies relevant to public health (including citizen protection and response, road safety health, infrastructure, human resources, etc.) are captured in Table 2. All respondents from government agencies indicated having relationships with multiple academic institutions as evidenced in their responses.

**Table 2:** Overview of decision-maker respondents

Government Level	Number of eligible decision-makers	Respondents (% of total)	Sex		Respondent agencies
			M	F	
City	20	6 (30)	1	5	Baltimore City Health Department [3] Baltimore City Board of Ethics [1] Baltimore City Department of Planning [2]
State	21	9 (42)	4	5	Maryland Department of Health [8] Maryland Department of Human Resources [1]
Federal	21	3 (14)	1	2	Health Resources and Services Administration [2] US Agency for International Development [1]
Global	30	6 (20)	2	4	World Health Organization [5] Global Alliance for Vaccine Initiatives [1]
Total (N)	92	24 (26)	8	16	

### Findings

Early in the interviews it became apparent that all faculty had much to share about engagement with decision-makers – regardless of our spill-over categorisations from Phase I as ‘engaged’ or ‘non-engaged’. Adhering to these measures risked not capturing our respondents’ past engagement that influence such engagement. We therefore discontinued use of the categories in our analysis and have reported all faculty responses in combination.

As we explored engagement experiences between faculty and decision-makers, we identified drivers for engagement or non-engagement that were critical precursors to initiation, maintenance and dissolution of these relationships. We therefore begin by describing the drivers for engagement.

#### Drivers for engagement

We identified 19 drivers that fell into 8 major categories, namely (1) decision-maker research needs, (2) learning, (3) access to resources, (4) student opportunities, (5) capacity strengthening, (6) strategic positioning, (7) institutional conditionalities, and (8) funder conditionalities. While political saliency and research relevance of a public health issue were the bedrock of the drivers we outline, we focus on those drivers that address the demand for and interest in relational interactions between academic researchers and decision-makers.

Table 3 outlines each of the 8 main drivers identified and provides quotes to support the 19 sub-categories within them. The table is colour coded to indicate prominence of engagement benefit. The legend can be found in the footnote of the table.

**Table 3:**
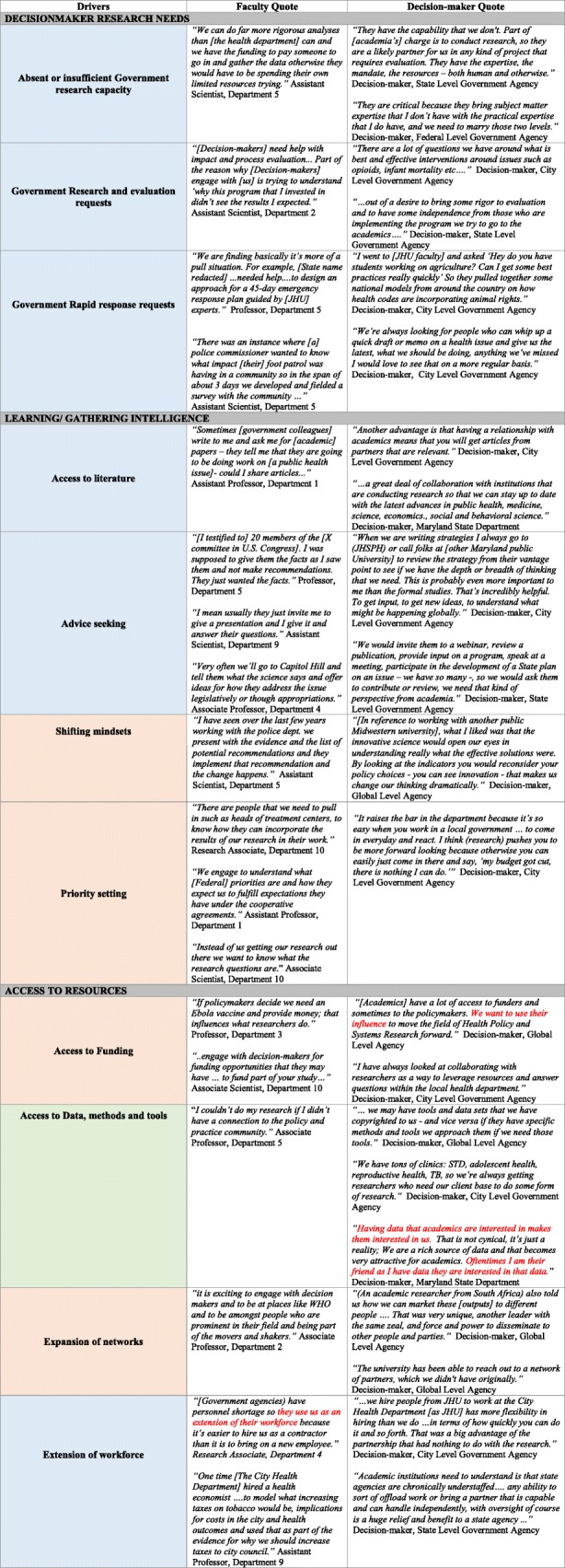

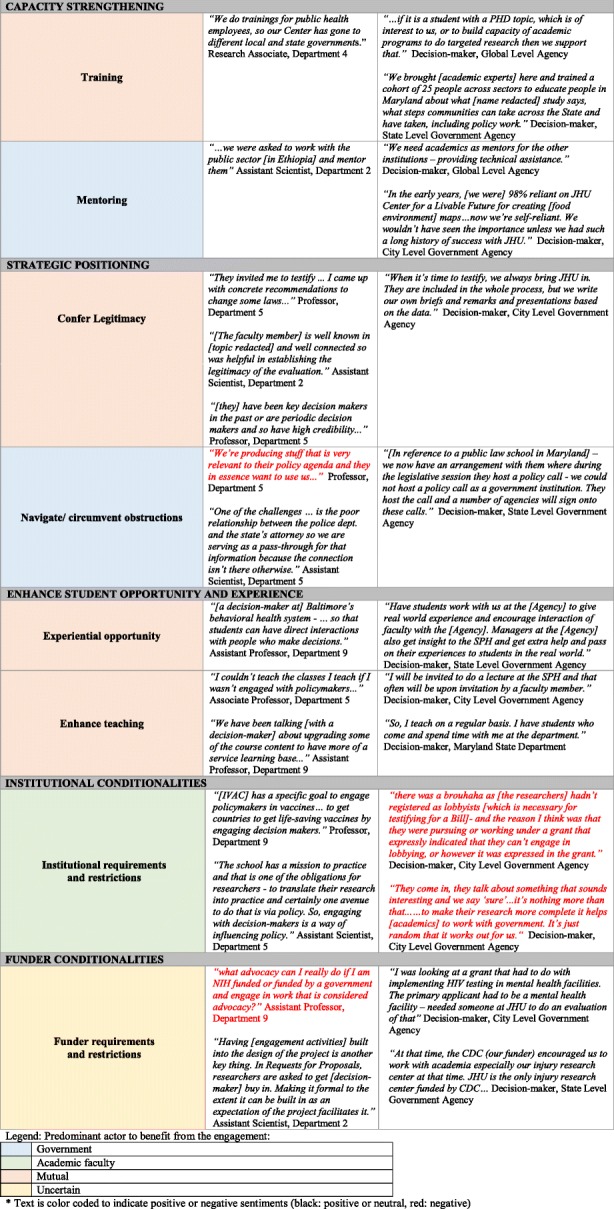
Drivers for engagement between academic faculty and government decision-makers

#### Engagement: why engage and who benefits?

Out of the 19 drivers, 8 appeared to primarily benefit decision-makers, 2 to primarily benefit researchers and 8 were mutually beneficial. External drivers of engagement, such as funder policies, indicated uncertainty as to who benefits.

Amongst drivers predominantly benefitting decision-makers, we found that perspectives of faculty as well as decision-makers echoed similar value and intention. For instance, ‘access to literature’ or ‘extension of workforce’ seemed to unidirectionally benefit decision-makers and this was confirmed by faculty as well as decision-maker respondents. This could perhaps be a result of assumptions of role as described here: “*If we are looking for deliverables and academic institutions are well positioned, we look at it as a service and they see it from a research perspective*” (Decision-maker, State Level Government Agency).

This suggests that the benefit may implicitly be mutual but more nuanced in its primary goal, particularly as we consider drivers such as ‘advice seeking’ or ‘mentoring’. However, as respondents described mutually beneficial drivers such as ‘expansion of networks’, ‘access to funding’ and ‘conferring legitimacy’, we heard examples of value that were unique to each party and not simply a concurrence of sentiments. For example, with respect to ‘priority-setting’ one faculty respondent emphasised that “*by engaging with decision-makers more so than policy-makers, you get a better sense of the context of what is going on … than being isolated from the real world*” (Associate Professor, Department 1)*.*

Complementing this, a decision-maker noted that “*my reason for going to meet researchers is … because I want to know what other State agencies are doing that is connected to the work that we are doing*” (Decision-maker, State Level Government Agency).

The most glaring difference however appears within the driver of ‘access to resources’ – and in particular ‘government-owned data’. This was raised by several decision-makers, particularly at the city and state level, as unidirectionally beneficial to academic researchers but did not appear in any of the faculty interviews. Federal and global level agencies were more likely to value and encourage integrated knowledge translation and co-production of research.

Experiences within which these various drivers were embedded suggest that, in many cases, the drivers were not mutually exclusive; several transpired in tandem, thereby reinforcing and supporting the overall rationale behind engagement. Multiple drivers for engagement uncovered different types and frequency of interactions between, oftentimes, the same parties. We explored these in the context of the stage of a relationship – initiation, maintenance and dissolution – as expounded below.

#### Relationship initiation, maintenance and dissolution

##### Individual initiation

There were several elements that enabled initiation of relationships by faculty and/or by decision-makers. These include the role of faculty in decision-making processes, individual attributes and reputation, institutional reputation, social capital, and the role of funders. Challenges such as contracting between organisations were also raised. Each of these are explored in more detail below.

Irrespective of the need to engage, we found that faculty and decision-makers’ desire to engage was embedded in their values, beliefs and perceptions of the role of faculty in contributing to decision-making problems and processes, through their expertise and research. This in turn affected their proclivity to initiate relations. From the faculty perspective, we heard a spectrum of views. Engagement and EIDM influence, for some faculty, trespasses the boundaries of a researcher’s role or desire: “*I don’t think it’s every researcher’s job to go and talk to a policymaker, I don’t think every researcher has the time or the inclination*” (Assistant Professor, Department 1).

For others, it appeared imperative – a moral and ethical obligation:

“*Science and policy are deeply intertwined. And to the extent that one wants to ensure prudent policy in your domain area … it’s our responsibility as academics, as scholars and presumably experts in our field to share insight and opinion …*” (Professor, Department 5)

“*When you’re wearing the JHU hat, you have a responsibility to evidence-based information and advocacy … I’m not here to have a lot of publications I am here to change public health for the better.*” (Associate Professor, Department 4)

Several Faculty also noted that, even if the desire exists, time and funding constraints determine whether or not they can initiate contact as expressed here:

“*We all have a lot of work to do and if* [engagement-related activities are] *not built into a grant or some other funding mechanism it can be hard to carve out time even if you do find it valuable.*” (Assistant Scientist, Department 5)

Decision-makers recognised that there are differences in how academics and government view the role of research as well as researchers. Many also highlighted that the context of decision-making is complex and perhaps abstract to those removed from it as expressed here: “*we have a unique charter and it limits legislative authority in some areas and you can’t expect a non-governmental person to know all this.*” (Decision-maker, City Level Government Agency).

However, the role of academia in decision-making processes was deemed vital amongst many respondents. This particular decision-maker provides an example:

“*Currently we have a legislator who has taken our recommendations from our report and is putting in front of legislature this year as a Bill.* [Faculty name redacted] *has been leading that work group and* [they] *will be testifying. It’s invaluable.*” (Decision-maker, State Level Government Agency)

We heard diametrically opposing views from faculty, though, on the role of decision-makers:

“*Most* [decision-makers] *I have been interacting with think research is important and when they invite me they care about how to use it to inform their results.*” (Assistant Scientist, Department 9)

“*Decision-makers should not be involved. They can set priorities and the way in which they apply these priorities is by funding basically. I frequently disagree with their priorities and I believe if they get involved in the process – it would be a train wreck. But this is for basic science...*” (Professor, Department 3)

Faculty as well as decision-makers confirmed that, once a need to engage has been identified, individual expertise and credibility is critical: “[Faculty name redacted] *has been working in this issue for 30 years so has a wealth of knowledge. When people are looking for experts, that is where they go.*” (Research Associate, Department 10)

This reputation is often driven by personal knowledge of individual academics or through a more public forum such as “*the national stage, presentations and being a national voice, media (interviews on NPR and news), all help build that reputation of those experts, so we know who they are.*” (Decision-maker, Federal Level Government Agency)

However, concerns about one’s reputation also arose, particularly if faculty felt that they were entering divisive territory as demonstrated in this statement: “*So, if you’re advocating for something that is quite controversial and there is not strong data on the side that you’re arguing, people can then call you an advocate. And being an advocate, for some people, is not an appropriate activity.*” (Professor, Department 5)

Several decision-makers noted, however, that advocacy is indeed important and wanted to reassure academics that registering as a lobbyist should not be considered unsavoury: “*It’s not a dirty term. It’s not any more of a dirty term than a lawyer is. Not anymore … pursuing lobbying means communicating with officials to promote a certain policy.*” (Decision-maker, City Level Government Agency)

The links between individual reputation and seniority of academic colleagues in being able to engender relations with decision-makers was only raised by faculty respondents. There was a perception that domestically as well as internationally: “*Senior, in terms of title and the way you look, matters. It comes with more credibility … I also think that senior people have had more time to build their networks.*” (Assistant Scientist, Department 2)

This is reflected in the composition of Task Forces or advisory boards as noted by faculty from several departments. In addition to individual reputation, several respondents highlighted the importance of institutional reputation as one that has afforded them opportunities and access that may not have otherwise been possible. They also recognise that:

“[Individual as well as institutional] *reputation works both ways and is a barrier in some cases: We’ve had some issues in some countries because of studies that were done a long time ago* [where colleagues] *came in and do the study and then take the data and leave.*” (Assistant Scientist, Department 2)

Some decision-makers also alluded to this by emphasising a need to exercise caution when choosing academic collaborators due to the implementation partners’ and community perceptions. Decision-makers noted that, often, institutional reputation rides on individual reputations. For instance, “*when you have had a good experience with one researcher at an academic institution, I am more likely to entrust to another person at the same academic institution.*” (Decision-maker, State Level Government Agency)

Historical relations, spanning over 15 years at times and predating individual faculty relations in some cases, were noted as another enabler. Others, as noted by faculty and decision-makers alike, were a result of personal alumni or student connections. Having well-connected colleagues or peers that broker introductions proved, oftentimes, formidable enablers for initiating relationships. Faculty respondents noted being introduced to decision-makers by department colleagues and leadership. Likewise, decision-makers relied on referrals from academic colleagues as demonstrated here: “[Faculty name redacted] *was on the Task Force … and was the conduit to my relationships at JHU … In the same way that she is my key person, I am also her key person.*” (Decision-maker, City Level Government Agency)

Leveraging intermediary connections such as donors and advocacy organisations to broker relationships was also noted frequently. From an operational standpoint, initiating projects with a private university like JHSPH proved challenging for many decision-makers who mentioned that,

“*The easiest way for the State is to engage with the university that is another State agency as it’s an easier procurement than going through a larger process that would allow* [private universities] *to bid* …” (Decision-maker, State Level Government Agency)

This has often resulted in preference for State universities as a first choice and JHSPH only if the needs of the government agency cannot be met otherwise. Furthermore, reliance on external funders to support engagement activities was something that one respondent suggested be absorbed institutionally:

“*We have a partner that we fund who provides a small stipend to … PhD students to teach them how to pull the policy relevant findings out of the research that they are doing. Maybe universities could organise that effort themselves.*” (Decision-maker, Federal Level Government Agency)

While one respondent from WHO expressed frustration that not all research is relevant for decision-making, faculty respondents noted that, “*you have to be able to have the money to be around and being part of such conversations where you get a sense of what is happening, knowing what you can do to meet* [decision-maker] *needs.*” (Associate Scientist, Department 2)

Access to such funding is particularly important as many city and state government respondents noted that they do not have the funds to support notably valuable collaborations.

##### Maintenance

There were several factors that enabled the sustainability of academic–government engagement. While saliency of the issue was noted as critical to establishing connections with decision-makers, the nature of the relationship was key to maintaining them. Transactional relationships appeared to occur, in most cases, when the drivers for engagement were predominantly unidirectional. For instance,

“ *… when we as a State are contracting with* [academic partners]*, our thought is: ‘you are a contractor for us, you are a vendor, you are providing a service’ – we have a specific deliverable or a need that we need to accomplish as a State.*” (Decision-maker, State Level Government Agency)

On the other hand, engagements that were more collaborative in nature include examples of co-production of research, joint publications or Op-Eds, co-teaching, and generally a mutually beneficial outcome. These were noted as having a better likelihood of prolonged engagement: “*The understanding that in order for it to be beneficial for both parties they have to have coincident gains.*” (Decision-maker, City Level Government Agency)

Having a champion who has a sense of ownership, drives the issue forward and leverages the established relationships was raised as an important factor in maintaining connections. One faculty expressed this aptly in their own reflection:

“*... I used to think that you just needed to do good strong science and that’s good, but you can do good strong science and it have no impact, so you really need somebody who sees the need and the importance of the research and will push it.*” (Assistant Scientist, Department 2)

Strong relationships with such individuals also assisted with traction on other issues. We had several examples where decision-makers rotated into different agencies that proved beneficial to faculty:

“*A good example is a relationship I’ve been cultivating with someone who worked in industry and is now at a federal agency. It just happens to be due to timing and that it fits well with the work I am doing that I now have a connection in the agency.*” (Associate Scientist, Department 4)

##### Dissolution

With emphasis often on creating and maintaining relationships, respondents described situations that result in the breakdown or dissolution of relationships. Many of these, in some way, were examples similar to those for maintenance except from a contrary perspective. For instance, reliance on a champion, while beneficial in some cases, was also noted as a hindrance to progress particularly in situations of retirement, opportunities for promotion, or when political cycles result in high staff mobility and turnover. Two examples are included below:

“*We have achieved buy in from key decision-makers and then there are elections and that person is no longer elected – this is something beyond your control. … most LMICs are not stable so making predictions is very difficult and there might be a coup and you’re done. Either start* [a new relationship] *from scratch or abandon the idea.*” (Research Associate, Department 2)

“ *… partnerships could be susceptible to falling apart because a lot of the times when* [staff] *leave, the institutional knowledge and oversight about the project can drift and the whole thing can fall apart.*” (Decision-maker, State Level Government Agency)

#### Pervasive elements

Finally, there were some engagement factors that were pervasive such as funding. Having non-research related funds to support engagement have often assisted with maintaining and nurturing relationships. City and state level agencies, however, noted that the contractual burden as well as oftentimes high overheads charged by universities pose a veritable challenge in engagement deliberations.

We found clear recognition that having professional/paid time to engage can help with both initiation as well as maintenance; however, establishing these opportunities could be restrictive, limiting or possibly invite bias into the engagement (depending on the funding source). Faculty members from various departments provide examples here:

“[Faculty member name redacted] *had a lot of admin support, he had funding to host lunch, those things make a difference for sure...*” (Associate Professor, Department 1)

“*I have reached out to the local government and hosted a day to share the findings and also get the input from the local chiefs and medical officers. These things cost money ….*” (Research Associate, Department 9)

“*Engagement was built in all stages* [of the grant proposal]*. It was formalised and was assumed that there will be mechanisms to engage*.” (Assistant Scientist, Department 2)

Another theme that emerged throughout the discussions was that mutual understanding of the implications of the research needed to be coupled with a relevant form of communication – a skill often raised as critical to these engagements:

“*You have to be able to describe things in ways that they understand and not in ways that make you sound like you are in an ivory tower.*” (Associate Professor, Department 9)

“*I think what is most helpful when there is an academic researcher can translate between what does a research finding mean – and kind of doing that translation about ‘this is what you can or cannot take from this research’ or really helping us walk through what it means.*” (Decision-maker, Maryland Department of Health)

In addition to the above, a myriad of other personal attributes such as mutual appreciation, respect, trust, humility and being responsive were found to contribute to all stages of academic–decision-maker relationships and assist in realising the gains mentioned earlier. These are noted below:

“*There is no mechanical process or magical formula.... it’s about personal relationships and trust. The more that you can build the trust and the more that you can respond to the needs.*” (Decision-maker, Global Level Agency)

“ *… we don’t publish a lot of things we do, and we don’t publish for a reason – it is to protect the trust that you build and the advice that you build and keep people coming back. The last thing that they want is that all of their conversation and insights go to a publication that the world sees. Or that it uncovers deficiencies that they don’t want people to know about and we respect that.*” (Senior Research Associate, Department 2)

“*The staff are really smart. If you go to Capitol Hill and think you’re going to patronize them and they’re going to roll over because you have a doctorate degree you’re not going to be very effective.*” (Associate Professor, Department 4)

“*You can’t say that you will get back to* [the decision-makers] *in a couple of days and then get back a week later … It’s not a great way of advertising yourself.*” (Decision-maker, Global Level Agency)

The findings demonstrate that faculty as well as government decisions harness relationships across sectors as well as institutions. This indicates a recognition that public health research, programmes and policies transcend health departments in order to engage a variety of stakeholders relevant to a public health concern. However, we were struck by the fact that most engagement appeared to be initiated by academics except when rapid responses for government decisions were required, for example, in crisis or urgent situations, or when there were little to no financial costs to engaging. Regardless of the maturity or history of relationships though, the majority of respondents acknowledged the need to navigate power dynamics, leverage intermediaries, and limitations to individual activities. This is a notable strength not only for the individuals but also for the institutions in which they are in.

#### Institutionalising relationships

While the above experiences revolve around individual relationships and personal attributes, institutional structures and supports were often reported to enable relationship sustainability, many of which evolved from lived experiences, as we describe below.

The importance of understanding the role of the institution in supporting academic–decision-maker engagements was emphasised by both faculty and decision-makers as being critical for the sustainability of engagement. The argument stemmed from the assertion that relationships are enhanced by faculty as well as decision-makers having a reciprocal appreciation of the partner’s context. This bidirectional experience has proven to be mutually beneficial as demonstrated in the quotes below:

“*I rotated in all the public sector organizations at local, state, federal and that was important to me to understand how all those levels work and in various stages in my career I may be able to liaise with each of those levels in public health …* ” (Instructor, Department 5)

“ *… Part of the benefit is that the researcher has a better understanding of who the people are within the State agency and what their needs are, what the policy and political landscape that affects that agency are … because those things can change so quickly that having academic partners that know what those things are and how they operate can make life a lot easier in terms of being flexible and how you navigate that over time ….*” (Decision-maker, State Level Government Agency)

Such ‘embeddedness’ can also help allay the scepticism that several decision-makers impressed:

“*We also look at people’s agenda...you know in order to maintain faculty status, you have to have published a certain number of publications, you have to have raised money for yourself through research grants, that is not the same as ‘we really want to improve this community’. It’s not that those two cannot be married to each other but because there is potential for perverse incentives, we have to look at that with a bit more rigor*” (Decision-maker, City Level Government Agency)

In recognition of this scepticism, one faculty respondent working in international settings highlighted that: “*We are a US based academic organization so there is always some level of suspicion* [internationally] *as to why you are doing these things and what you are going to do with the information.*” (Senior Research Associate, Department 2)

## Discussion

Gordon [54] asserted that “*if the perceived distance between the goals of public health agencies and the goals of schools of public health has narrowed, it is the interaction of individual faculty members and public health professionals who are responsible for bridging the gap*”. However, several years later, the Health Resources and Services Administration [73] reported that, “*while there are a few examples of successful collaborations between schools of public health and public health agencies at the local level, schools of public health, in general, have done a poor job of partnering with these agencies*”.

Our results demonstrate that many engagements are dynamic and result from historic and mutually respectful relationships between SPH faculty and government decision-makers. We began with examining the drivers for engagement and unpacking how these drivers affect the initiation, maintenance and/or dissolution of relationships. Importantly, we examined those elements that played a role in each stage of relationship evolution as well as those that permeated throughout. We did not however seek to interrogate the impact of these relationships on policy influence.

We detail three overarching areas of discussion below – the nuances of our results and their implications for higher education institutions (HEIs) as well as government agencies in the quest for EIDM, our reflections on how our results are situated within existing frameworks that seek to classify relationships, and finally the strengths and limitations of our study.

### Implications for HEIs as well as government agencies in the quest for EIDM

#### The interplay between engagement drivers, partnership evolution and social networks

We found 19 drivers that fell into 8 major categories. In reviewing other frameworks for engagement drivers, we note some alignment with those described in other contexts [27]. In addition to the drivers, we also find similarities in the characteristics of enduring relationships experienced by our respondents with that of others [74–76]. However, when we consider drivers for engagement in the context of relationship enablers, we find a unique interplay between how the ‘motivation, the need, the desire, or the context’ to engage (the drivers) interacts with ‘who’ to engage with (the people) and how that evolves (the journey).

Regardless of the driver(s) that encouraged engagement between academic researchers and government decision-makers, the choice of who to engage was almost always determined by individual social capital. Similar to our findings, Hamilton [77] also notes the importance of “*the origin of collaboration*” stemming, at times, from individual initiative. While a faculty member’s scientific and technical human capital [78] were the door to entry, their social capital – alumni connections, past engagements and/or referrals – were often the password to that very door. Similarly, while geographical distance between academics and decision-makers often times determined the ability to engage, the multiplexity and history of relationships – ‘the remembered past’ – dominates not only the willingness and desire to engage [69, 79] but also the resilience of those engagements [80, 81]. Some unexpected and uncontrollable factors, however, can affect the survival of relationships as well as their evolution. For instance, similar to other global contexts, staff turnover and posts remaining unfilled for significant periods of time create a veritable challenge to initiating or maintaining relationships and is often the biggest reason for partnership dissolution [20, 69, 82–84].

Therefore, the power of individual relations cannot be overstated. These relations need to be recognised, mapped, analysed, harnessed and leveraged responsibly [47, 69, 85], keeping in mind that power can be beneficial as well as detrimental to partnerships and collaborations [77]. While it is expected, and indeed accepted, that not all those engaged in the research process will benefit equally from it [86], we seek to reflect on the imbalance of who appears to benefit most from these engagements versus who primarily takes responsibility for maintaining them. Ross et al. [87] assert that the funding environment that either supports or hinders engagement results in decision-makers either providing formal support, being a responsive audience or serving as an integral partner. We would expand this to include decision-makers being acquiescent or reticent partners in some cases as deduced from our interviews, particularly when benefits, while clear, are perceived to manifest much later.

Action to determine what comprises ‘benefit’ as well as methods to recognise and capture these in meaningful ways [88] would go a long way in diffusing negative perceptions. Furthermore, a more deliberate stakeholder analysis that seeks to understand varying levels of benefit for stakeholders with differing levels of interest in the research (or policy) issue would be important to consider [43]. Boaz et al. [89] provide some design principles to support improvement in stakeholder engagement with research.

#### Institutional and systemic structures

It is almost impossible to de-link who initiates a partnership and how from the why – the drivers. However, the dynamics of maintenance depend on institutional supporting structures such as institutional culture, leadership and support – moral as well as financial – rather than solely individual capital, particularly outside of grant-required engagements.

The prevalence of multiplex engagements rather than one-off partnerships appeared to be a key strength of many of the relationships suggesting the importance of more systemic arrangements. These can manifest in various ways such as temporary transfers or secondments [90], dedicated policy engagement coordinator positions, policy buddies [91] and deliberative dialogues [61, 92, 93], amongst others. Olivier et al. [94] provide several more examples as well as outline some of the characteristics, benefits and challenges of such embeddedness within the field of health policy and systems research.

The emphasis on establishing institutional structures to overcome the challenges of dependence on individuals [37, 68, 95] as well as short-term funding cycles [77, 96] suggests that the SPH (and other HEIs) as well as government agencies should consider multiplex engagements by enhancing existing institutional strategies, considering other documented strategies, and innovating with new ideas relevant to the context, particularly with respect to funding engagement activities and processes.

### The heterogeneity of responses within and between government and academic faculty respondents

It is not surprising that the most rewarding relationships described were those where the research project was co-conceived, where collaborations were clear, and where there was mutual benefit [27, 72, 87, 97, 98]. However, the perceptions of whether this falls within the purview of academics, particularly early career researchers, varied across departments in the SPH. Similar to previous work [69, 99–101], it appears that individual value, motivations, attributes and experiences supersede departmental or institutional structures to engage. The confluence of all these may explain some of the divergences in how SPH faculty respond to opportunities to engage with decision-makers [102]. Several of the responses demonstrate contradictions and indeed even tensions in the transformational role of universities or academics. As Brennan et al. [2] note, “*similar tensions exist in other places (within the university) and should serve as a reminder that universities contain many contradictions, even at a single point in time*”.

Similarly, we found that perceived benefits as well as pitfalls of engagement with academia varied across government levels. This is likely compounded by the fact that Federal and global level agencies play a dual role of funder as well as decision-maker unlike government officials at the city and state levels, where the lack of funding for research oftentimes hinders research priority-setting, support and commissioning. In the quest for equitable partnerships and more EIDM, it would behove researchers to appreciate this complexity.

### The impact of funder imperatives

Shifting funder requirements for research, such as required partnerships, demonstration of social return on investment and more effective means of integrated knowledge translation [6, 103–108] have clearly influenced the evolution of some of the relationships between the SPH and government agencies and influenced policy-focused public health research [94, 109]. However, challenges persist when operationalising some of these with restrictions and varied interpretations of engagement parameters. While funder requirements seek to encourage more socially focused returns on research endeavours, there are veritable concerns that this could also lead to perverse incentives resulting in activities that lead to easily quantifiable instrumental impacts [110, 111]. Furthermore, varying terminologies, for instance, with respect to activism, advocacy and lobbying, between government [112, 113] and academia [114–116] that appear to hinder engagement, need to be confronted if indeed government and academia seek to collectively influence decision-making.

City or state partners funded by Federal agencies in the United States are often required to first contract with public institutions. Universities such as the SPH in this study are therefore at a contractual disadvantage due to their designations as ‘private’. A clear understanding of the technical and reputational benefits of engaging with each ‘private’ institution weighed against the transactional and administrative costs is imperative in order to justify choice of partners as well as the additional complexities of engagement [77]. Private academic institutions must also recognise this reality and consider ways to reduce the contractual and financial burden for government agencies if they wish to be considered partners of choice. Guidance on how to navigate this space can be found through a number of organisations in the United States [112, 117, 118].

We summarise the main issues highlighted in this discussion in Table 4, together with relevant recommendations and implications for academia as well as government agencies.

**Table 4:** Salient issues, recommendations and implications for the evolution of academic–government relationships

Issue	Recommendations		Implications
	Academia	Government	
Irrespective of who benefits from a partnership, the responsibility for initiating engagement often falls upon academia	• Embrace this reality and foster strategies to engage that are effective, efficient, genuine and resilient • Diversify the range of contributions in order to anticipate, respond to government needs (e.g. individual research projects, syntheses of research, rapid response, testimony, technical and advisory boards etc. …)	• Better understand the capacity that exists in academia to serve specific needs • Diversify methods of outreach for evidence needs in a way that academia can respond to (i.e. that go beyond traditional calls for research proposals if indeed rapid response services are required) • Clarify and provide processes required for partners to engage in such activities • Take more initiative to engage with academia	Innovation in collaborations and increased relevant engagement will • permit universities to enhance policy understanding and shed their ‘ivory-tower’ image while maintaining ‘protected space’ for critical thinking • allow government agencies to enhance their ‘evidence-informed’ culture
Perceptions of transactional relationships persist	• Ensure that the goals and intended impacts of the partnership are explicit • Establish more systemic and transformative systems of engagement in contrast to issue-based (perceived) transactional relationships • Expand the interpretation of relationship benefits to transcend quantifiable and tangible outputs to include unquantifiable but valuable outcomes such as learning, relationship building and capacity enhancement • Recognise and capture intangible outcomes in meaningful ways		Mutually beneficial transformational partnerships will become the norm, allowing negative perceptions to diffuse over time
Dependence on individual relationships can be a strength as well as a weakness in initiating, maintaining and terminating relationships	• Recognise, map and analyse existing relationships for their potential to advance/hinder potential collaboration • Instil strategies to mitigate negative effects of agency staff/faculty turnover • Strengthen the breadth, depth and diversity of relationships between academia and government • Build relationships with a variety of actors that can serve as intermediaries or brokers • Combine individual drivers with institutional structures to support the cultivation of relationships • Consider multiplex engagements by enhancing existing institutional strategies, considering other documented strategies and innovating with new ideas relevant to the context particularly with respect to funding engagement activities and processes		Multi-actor, multiplex and multisectoral relationships will permit health-focus institutions to impact health from a variety of angles as envisaged by the Sustainable Development Goals
Not all parties are willing and/or able to foster relationships with each other	• Understand the engagement complexities or challenges faced by potential partners • Encourage a spectrum of opportunities for faculty or staff willing to engage • Provide the necessary (moral, professional, technical and financial) support structures as well as time required to enable this		If benefits to a relationship are seen not only in the form of tangible outputs but also in terms of perhaps unquantifiable but valuable outcomes, then we may see an increase in willingness to engage Enhanced support to the engagement-inclined will likely lead to increased ability to cultivate meaningful and long-lasting individual as well as institutional relationships
	• Pay attention to early career academics who are particularly disadvantaged given efforts required for academic advancement • Provide a coordinated approach and response to government for student practical needs	• Diversify staff profiles by recruiting and/or encouraging the hiring of decision-makers with academic backgrounds • Provide a coordinated approach and response to academia for student practical opportunities	
Varied use and interpretation of terminology, particularly for advocacy and lobbying, results in misunderstanding as well as structural barriers to engagement	• Leverage partnerships with advocacy organisations as key knowledge brokers between academia and government		Managed expectations, processes and implications of ‘political’ engagements will reduce uncertainties and misunderstandings Clearly articulated boundaries will temper hesitancies as well as protect activities from external scrutiny
	• Expand beyond defining terminology to providing examples of what, within each type of activities, is permissible and prohibited by faculty • Assist government with better understanding these decisions and their impact on faculty activities	• Expand beyond defining terminology to providing examples of activities that constitute advocacy and lobbying • Clarify and provide processes required for partners to engage in such activities (e.g. need to register as a lobbyist to give testimony etc.)	
Private universities such as the school of public health in this study may face contractual challenges with government agencies	• Capitalise on academic rigor, expertise, reputation networks and relationships to justify the additional complexities of engagement • Consider ways to reduce the contractual burden for government • Reassess the proportionate overhead being charged to collaborations with government agencies, particularly at the city and state levels • Explore more complementary partnerships with public Universities • Assist government agencies with better understanding the processes (documents, timeliness, steps, costs, flexibility, etc.) for contracting with the school of public health	• Recognise the variety of benefits as well as costs of engaging with private versus public universities • Reconsider the weighting of ‘costs’ (contracting, overheads, etc.) versus ‘benefits’ (academic rigor, issue expertise and reputation) in selecting a partner of choice • Explore partnerships with third-party organisations who may have fewer barriers to contract with private institutions • Incorporate recognition of the differences between engaging with faculty at a private versus public institution • Define and exemplify proper and improper engagements	Creating a shared understanding of the benefits and drawbacks associated with academics in public versus private universities will help decision-makers initiate more applicable engagements Accommodating the contractual realities of government agencies will allow academia to maintain a competitive advantage

### Situating our results in the context of current frameworks classifying relationships

Kothari et al.’s indicators [42] suggest that partnerships be classified and assessed based on the temporal maturity of the partnerships (not relationships). While this makes intuitive sense, we were challenged with the above given the experiences of our respondents who, while forging new professional partnerships at times, were capitalising on an established personal and social capital that brokered relationships for those partnerships. We therefore posit that ‘early’ and ‘mature’, as described by Kothari et al., reflects a dimension of time alone which we believe likely mis-categorises relationships built on previous (potentially indirect) foundations of strength or weakness. It also hinders the consideration of the social interaction model [98], which considers relationship dynamics based on the stage of the research process – knowledge production, dissemination and utilisation – given that not all partnerships need to, want to or are able to involve all parties at all stages of the process. We posit, similar to Provan [81], that, contrary to intuition, “*informal, interpersonal ties indicate an advanced, rather than an early level of network evolution*”, particularly in cases where academic researchers have experience in policy and practice settings [119] and vice versa. However, that evolution is either facilitated or hindered by factors that impact the initiation, maintenance and dissolution of relationships.

Others suggest classifying partnerships based on the frequency of interaction: infrequent, intermittent or recurrent [120–123]. In this study, where partnerships were sought primarily for one reason – ‘learning’, for instance – and/or were considered unilaterally beneficial, the interactions appeared to be infrequent or intermittent. Conversely, in situations spurred by several drivers for engagement, we noted the likelihood of more protracted or recurrent engagements that drew on each other for various reasons at various times. Additionally, previously fruitful relationships with individual faculty and/or their institutions significantly increased the likelihood of recurrent government agency collaborations. Therefore, taking due consideration of the factors that enhance initiation and maintenance of relationships between academic faculty and government agencies, efforts will likely generate long-term benefits in the form of relevant research and policy outcomes, steady or increasing levels of funding [27], group level identity transformation [124], and greater opportunities for student as well as faculty career experiences.

## Strengths and limitations

SPHs and public health programmes may manifest differently in other parts of the world with accreditation requirements as well as determination of what a SPH consists of likely to vary from country to country. For instance, in some parts of the world, public health training is embedded in faculties of health sciences [125] or departments of global health [126] or within schools of nursing and/or medicine [127]. Therefore, the context of our study, while relevant to institutions supporting public health, may not be transferable [128] to all contexts. Although the contextual complexity that surrounds any individual relationship is unique and likely not replicable, this study has uncovered some key elements that may well transcend the study context and thereby inform university faculty as well as government agencies more broadly as they seek to advance EIDM. The diverse perspectives within and between institutions included in the study provide a variety of insights that can be considered more broadly relevant.

The use of different modes for interviewing (video Skype, telephone) allowed us to expand reach to global decision-makers as well as those not located in close proximity to the SPH in this study. However, we recognise that there may have been variations in interviewer or respondent comfort between these modes. Furthermore, the change in the political administration in the United States in 2016 led to large-scale turnover of political appointees and government officials. Hence, limited access to public listing of personal contact information, changes in position or organisation and seniority of decision-makers resulted in invitations being sent to 69 decision-makers rather than the initial list of 92. We also note that our decision to use network analysis metrics from Phase I to select respondents for Phase II may have resulted in potentially insightful participants being excluded.

## Conclusion

Relationships between academic research faculty and government decision-makers are driven by various factors. The decision about who to engage with once the reason to engage has been established depends oftentimes on individual social capital and historical relations. However, the manner in which such relationships evolve is subject to individual, institutional, networked and systemic dynamics – initiation seems to depend somewhat on the drivers for engagement but is dominated by individual academic faculty. Maintenance relies on individual as well as institutional systems of support. Dissolution appears to rely on the former two as well as external unexpected or uncontrollable factors. Some conditions require pervasiveness while others require intermittent contributions. Irrespective of the conditions for collaborations and partnerships, the relationships need to comprise mutual benefit, respect, consideration and a long-term vision given the likelihood of multiplex interactions.

As universities and government agencies establish relationships with each other and utilise these opportunities to share ideas, envision change together, and leverage their collaborations to use evidence to inform decision-making, a new modus operandi becomes possible. Mutual learning – about contexts, processes, strengths and limitations of the collaborating organisations – can lead to new practices, alternate approaches and transformative change due to this interaction. Government agencies, SPHs and HEIs, more broadly, should consider the various drivers (motivations, reasons) for as well as enablers (mediators or elements that enhance or hinder) of relationship building and resilience in order to promote more EIDM.

## Data Availability

The datasets used and/or analysed during the current study are available from the corresponding author on reasonable request.

## Abbreviations

CEPH: Council on Education in Public Health
EIDM: Evidence-informed decision-making
HEIs: Higher education institutions
JHSPH: Johns Hopkins Bloomberg School of Public Health
SPH: School of public health
